# Nuclear imaging does not have clear added value in patients with low a priori chance of periprosthetic joint infection. A retrospective single-center experience

**DOI:** 10.5194/jbji-7-1-2022

**Published:** 2022-01-06

**Authors:** Karsten D. Ottink, Stefan J. Gelderman, Marjan Wouthuyzen-Bakker, Joris J. W. Ploegmakers, Andor W. J. M. Glaudemans, Paul C. Jutte

**Affiliations:** 1 Department of Orthopaedics, University of Groningen, University Medical Centre Groningen, Groningen, the Netherlands; 2 Department of Medical Microbiology and Infection Prevention, University of Groningen, University Medical Centre Groningen, Groningen, the Netherlands; 3 Department of Nuclear Medicine and Molecular Imaging, University of Groningen, University Medical Centre Groningen, Groningen, the Netherlands

## Abstract

**Background**: A low-grade periprosthetic joint infection (PJI) may present without specific symptoms, and its diagnosis remains a challenge. Three-phase bone scintigraphy (TPBS) and white blood cell (WBC) scintigraphy are incorporated into recently introduced diagnostic criteria for PJI, but their exact value in diagnosing low-grade PJI in patients with nonspecific symptoms remains unclear. **Methods**: In this retrospective study, we evaluated patients with a prosthetic joint of the hip or knee who underwent TPBS and/or WBC scintigraphy between 2009 and 2016 because of nonspecific symptoms. We reviewed and calculated diagnostic accuracy of the TPBS and/or WBC scintigraphy to diagnose or exclude PJI. PJI was defined based on multiple cultures obtained during revision surgery. In patients who did not undergo revision surgery, PJI was ruled out by clinical follow-up of at least 2 years absent of clinical signs of infection based on MSIS 2011 criteria. **Results**: A total of 373 patients were evaluated, including 340 TPBSs and 142 WBC scintigraphies. Thirteen patients (3.5 %) were diagnosed with a PJI. TPBS sensitivity, specificity, and positive and negative predictive values (PPV, NPV) were 71 %, 65 %, 8 % and 98 %, respectively. Thirty-five percent of TPBS showed increased uptake. Stratification for time intervals between the index arthroplasty and the onset of symptoms did not alter its diagnostic accuracy. WBC scintigraphy sensitivity, specificity, PPV and NPV were 30 %, 90 %, 25 % and 94 %, respectively. **Conclusion**: Nuclear imaging does not have clear added value in patients with low a priori chance of periprosthetic joint infection.

## 1 Introduction

The diagnosis of chronic periprosthetic joint infection (PJI) remains a challenge in modern orthopedics. This is especially true in patients who present with only pain or discomfort of the arthroplasty joint (Goswami et al., 2018). A low-grade infection, amongst other causes (such as aseptic loosening), could be the cause of the symptoms and has to be diagnosed or excluded as it has serious consequences for subsequent treatment (Romanò et al., 2017; Parvizi et al., 2014; Yoon et al., 2017). However, in case of a low-grade infection, clear signs and symptoms of infection are often absent, and serum marker tests are usually nonspecific or conflicting (Pérez-Prieto et al., 2017; Schiffner et al., 2019). Synovial fluid markers, microbiological cultures and histology may aid in diagnosis but require invasive procedures (Ottink et al., 2018, 2019; Wouthuyzen-Bakker et al., 2018). Still, even with all diagnostic modalities available, diagnosis of low-grade PJI remains a challenge (Jutte et al., 2014; Parvizi et al., 2018; Sconfienza et al., 2019). Furthermore, it is not unusual to have a punctio sicca, especially in arthrocentesis of the hip, depriving one of these synovial fluid markers.

Among the different diagnostic criteria available for PJI diagnosis (Signore et al., 2019), the World Association against Infection in Orthopaedics and Trauma (WAIOT) (Romanò et al., 2019; Bozhkova et al., 2020), the European Association of Nuclear Medicine (EANM) and the European Bone and Joint Infection Society (EBJIS) (McNally et al., 2021) have incorporated nuclear imaging into their diagnostic criteria. The WAIOT and EBJIS PJI criteria use technetium-99m-methylene diphosphonate ( ${}^{99m}$ Tc-MDP) three-phase bone scintigraphy (TPBS) as a “rule-out” test and ${}^{99m}$ Tc-labeled white blood cell (WBC) scintigraphy (sometimes combined with a bone marrow scan) as a “rule-in” test (Romanò et al., 2020).

The evidence supporting nuclear medicine in PJI diagnosis was critically appraised by Verberne et al. (2016, 2017), who concluded that the quality of the studies was mediocre and that they had mostly been performed on patients with a high probability of PJI, causing bias (Glaudemans et al., 2013; Blanc et al., 2019; Teiler et al., 2020). One study evaluated its diagnostic accuracy in patients with a lower probability of PJI (Trevail et al., 2016).

Therefore, the purpose of this retrospective study is to evaluate the diagnostic value of TPBS and WBC scintigraphy in patients with nonspecific symptoms in which a low-grade PJI was part of the differential diagnosis.

## 2 Methods

### 2.1 Patient cohort

During the period from 2009 to 2016, all patients with a symptomatic arthroplasty joint who underwent a TPBS and/or WBC scintigraphy were included. Patient selection was done by cross-referencing all patients who underwent one of these scans and who received a primary arthroplasty and/or a subsequent revision arthroplasty in the University Medical Centre Groningen (UMCG) in the Netherlands. Exclusion criteria were (i) patients with clear signs of infection such as the presence of a sinus tract or the onset of acute symptoms and signs of an infection, (ii) patients with a tumor prosthesis and (iii) patients who received nuclear imaging within 3 months after the index surgery.

The diagnostic protocol was in accordance with the 2011 MSIS criteria and conforming proven practice from 2009 until 2011 and consisted of the evaluation of patient history, physical examination, plain X-rays, and blood-serum inflammatory markers (Parvizi et al., 2011). If there was any doubt whether a low-grade PJI could be present based on these determinants, a TPBS and/or WBC scintigraphy was performed. Additional arthrocentesis of the joint was performed for synovial fluid markers and culture, when a PJI could not be excluded based on prior non-invasive diagnostics. In specific cases, multiple soft tissue biopsies were acquired for microbiological culture and histological analysis.

The 2011 MSIS PJI diagnostic criteria consist of the following.

1. Presence of a sinus tract (excluded in this study)
2. Pathogen is isolated by culture from at least two separate tissue or fluid samples obtained from the affected prosthetic joint.
3. Four of the following six criteria exist.
  a. Elevated serum erythrocyte sedimentation rate (ESR) and serum C-reactive protein (CRP) concentration
  b. Elevated synovial leukocyte count
  c. Elevated synovial neutrophil percentage (polymorphonuclear (PMN) percentage of leukocytes)
  d. Presence of purulence in the affected joint
  e. Isolation of a microorganism in one culture of periprosthetic tissue or fluid
  f. More than five neutrophils per high-power field in five high-power fields observed from histologic analysis of periprosthetic tissue at 9400 magnification

Data collection consisted of all determinants mentioned above. The time between the index arthroplasty surgery (primary implantation or revision arthroplasty) and the onset of symptoms was determined. The use of antibiotics and immune-suppressive drugs (e.g., antirheumatics) at the time of diagnostic test was noted as these could influence the process of diagnosing a PJI.

### 2.2 Image acquisition and interpretation

According to the applicable international guidelines at that time, patients who underwent TPBS received approximately 700 MBq ${}^{99m}$ Tc-HDP intravenously. Phase 1 (perfusion phase) started immediately after the injection and lasted 2 min. The second phase (diffusion phase) started at the second minute until the fifth minute. The third phase started 3 h after the injection in which a static image was taken in anterior and posterior positions. TPBS was considered negative if no increased focal uptake was present in all three phases near the prosthesis in comparison to other bones (background). In case of a present infection, a single-photon emission computerized tomography and X-ray computed tomography (SPECT/CT) scan was acquired for the exact location. In general, no further imaging was advised in cases with a negative bone scintigraphy, but an additional WBC scintigraphy was recommended in cases with a positive TPBS.

In patients who underwent a WBC scintigraphy, 50–100 cc of blood was collected, and the white blood cells were labeled with 370–550 MBq ${}^{99m}$ TC-hexamethylpropyleneamine oxime (HMPAO) (de Vries et al., 2010). The labeled autologous white blood cells were then re-injected, and two images were taken with acquisition time corrected for decay. The first image was acquired 2–4 h and the second image 20–24 h after re-injection. The image was considered positive for infection when there was accumulation of leucocytes that increased in intensity or size over time. In case of positivity, a SPECT/CT scan was performed for the exact location of the infection. It must be remarked that this protocol was implemented in 2014 (Glaudemans et al., 2013). Before that time point, images were acquired with a fixed number of counts at the same time points (El Espera et al., 2004).

In this study we used the conclusion of the nuclear medicine physician as stated in the reports of the TPBS and WBC scintigraphy in the electronic patient files to define whether infection was suspected or not.

### 2.3 Reference standard

PJI was based on intra-operative cultures when revision surgery was performed. During revision surgery at least five tissue cultures were obtained. PJI was diagnosed if at least two cultures were positive with the same microorganism according to the MSIS criteria. However, in case virulent microorganisms were detected, one positive culture sufficed (i.e., *Staphylococcus aureus*, Gram-negative rods, *Candida*, and *Enterococcus* species) in addition to another minor criteria (MSIS criteria). In patients who did not undergo revision surgery, PJI was ruled out by clinical follow-up of at least 2 years if there were no signs of infection. The MSIS diagnostic criteria for PJI from 2011 were used in our clinic at that time, and before 2011 the same criteria were already applied (Parvizi et al., 2011).

### 2.4 Statistical analysis

The diagnostic accuracy of nuclear imaging was calculated (sensitivity, specificity, positive predictive value (PPV), negative predictive value (NPV), positive likelihood ratio (PLR), negative likelihood ratio (NLR) and the diagnostic odds ratio).

According to the recommendations made in the EBJIS definition criteria, stratification was performed for TPBS with arthroplasties less than 2 years, more than 2 years, and more than 5 years after implantation, and sub-analyses were performed for hips and knees separately (Romanò et al., 2019; Niccoli et al., 2017).

Since the currently recommended image acquisition and interpretation protocol for WBC scintigraphy was incorporated in January 2014, stratification of the two cohorts was performed to rule out potential confounding.

## 3 Results

### 3.1 Patient population

From 2009 to 2016, 291 patients with a primary arthroplasty and 82 with a revised arthroplasty were included in this study (total 373). Patient demographics are shown in Table 1. The indications for the primary and revision arthroplasties (index surgery) are shown in Table 2. Thirteen out of the 373 cases were eventually diagnosed with a PJI based on intra-operative cultures (3.5 %). In the group of patients who did not undergo revision surgery, no infections were diagnosed during 2-year follow-up. Two patients were treated with antibiotics before and during the revision surgery, and 18 patients used immune-suppressive drugs (such as tumor-necrosis-factor α inhibitors, methotrexate, prednisolone, and disease-modifying antirheumatic drugs). None of the patients using antibiotics or disease-modifying antirheumatic drugs (which could hinder the diagnosis of PJI) were diagnosed with a PJI during 2 years of follow-up.

**Table 1:** Demographics.

Baseline data	n	
Number of patients	373	
Median age during implantation in years (range)	60.5	(20.4–86.4)
Male/female (%)	132/241	(35 %/65 %)
Total hip arthroplasty (%)	223	(60 %)
Hip hemi-arthroplasty (%)	5	(1 %)
Total knee arthroplasty (%)	134	(36 %)
Hemi knee arthroplasty (%)	11	(3 %)
Cemented/uncemented (%)	245/128	(66 %/34 %)
Primary/revision arthroplasty (%)	291/82	(78 %/22 %)
Median time onset of symptoms after index surgery in months (range)	40	(6–19.0)
Median serum CRP level (range)	4.6	(0.3–75)
Median serum CRP mg/L (range)	4.6	(0.3–75)
Median serum leucocyte count X 109/L (range)	7.2	(1.4–16.0)
Number of TPBS	340	
Number of WBC scans	142	
Number of combined TPBS and WBC scans	109	
Number of PJIs diagnosed by the reference standard	13	(3.5 %)
Average time between index tests and the reference standard in months (95 % confidence interval)	9.3	(8.6–10.1)

**Table 2:** Indication of primary and revision arthroplasty.

Cohort	Diagnosis	n	%
Primary	Osteoarthritis (OA)	217	75 %
arthroplasty	Secondary OA – posttraumatic	15	5 %
cohort	Secondary OA – inflammation/rheumatoid arthritis	11	4 %
( n=291)	Secondary OA – avascular necrosis femoral head	20	7 %
	Secondary OA – developmental of hip dysplasia	3	1 %
	Secondary OA – iatrogenic	5	2 %
	Hip fracture	16	5 %
	Fracture pseudo-arthrosis	2	1 %
Revision	Aseptic loosening of the arthroplasty component	48	59 %
arthroplasty	Instability/malposition	14	17 %
cohort	Periprosthetic joint infection	9	11 %
( n=82 )	Residual osteoarthritis: hemi knee or patella	7	6 %
	Increased cobalt level in blood serum	1	1 %
	Periprosthetic fracture	3	6 %

From the patients diagnosed with a PJI, the causing microorganisms were *Cutibacterium acnes* ( n=4 , 1 as second culture), *Staphylococcus epidermidis* ( n=3 ), *Staphylococcus capitis* ( n=3 ), *Streptococcus mutans* ( n=1 ), *Corynebacterium* spp. ( n=1 ), *Parvimonas micra* ( n=1 ) and *Pseudomonas aeruginosa* ( n=1 ).

### 3.2 Bone scintigraphy

A total of 340 TPBSs were performed: 217 were true negative and 4 false negative, resulting in a NPV of 98 % (Table 3). Thirty-five percent of the TPBS ( n=119 ) showed an increased uptake, but because TPBS is not able to differentiate a PJI from other causes, these positive reports were considered inconclusive from a clinical point of view. The corresponding sensitivity, specificity and PPV were 71 %, 65 % and 8 %, respectively. Stratification for time intervals between the index arthroplasty and the onset of symptoms or the type of joint (hips and knees) did not alter its diagnostic accuracy (Table 3).

**Table 3:** Diagnostic accuracy of TPBS stratified for different cohorts for time of onset of initial symptoms after index surgery.

Estimates from the observed sample	Complete	<2 yr	>2 yr	>2 yr	>5 yr	>5 yr
Time of onset of symptoms after index surgery	cohort			THA only		TKA only
Number of patients in the cohort	340	149	191	129	123	34
Number of patients with increased uptake on bone scan	119	60	59	32	32	13
Sensitivity	71 %	60 %	50 %	67 %	50 %	0 %
Specificity	65 %	60 %	70 %	74 %	71 %	61 %
Positive predictive value	8 %	5 %	3 %	6 %	6 %	0 %
Negative predictive value if a PJI is clearly excluded	98 %	98 %	98 %	99 %	98 %	95 %
Diagnostic odds ratio	4.6	2.3	2.3	5.8	2.5	0.0
Positive likelihood ratio	2.0	1.5	1.6	2.6	1.8	0.0
Negative likelihood ratio	0.4	0.7	0.7	0.4	0.7	1.7

### 3.3 WBC scintigraphy

**Figure 1:**
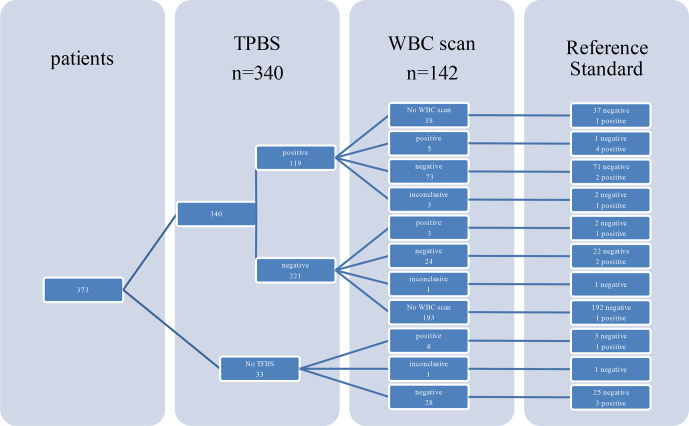
Flowchart made of nuclear imaging.

A total of 142 WBC scintigraphies were performed (Table 4). WBC scintigraphy was negative in 125 patients, 7 of which were false negative. WBC scintigraphy was positive in 12 patients, 9 of which were false positive. Five WBC scintigraphy reports were inconclusive. WBC scintigraphy sensitivity, specificity, PPV and NPV were 30 %, 90 %, 25 % and 94 %, respectively. As depicted in Fig. 1, in 108 patients a WBC scintigraphy was performed after a preceding TPBS. Twenty-eight WBC scintigraphies were made after a negative TPBS and 80 WBC scintigraphies after a positive TPBS. The combination of both scans did not increase the diagnostic accuracy compared to the WBC scintigraphy alone (Table 4).

**Table 4:** Diagnostic accuracy of WBC scintigraphy and combination of TPBS/WBC scan for the diagnosis of a low-grade PJI.

Estimates from the	WBC	Cohort WBC	WBC	WBC	Diagnostic
observed sample	scintigraphy	scintigraphy with	scintigraphy	scintigraphy	protocol
	cohort	culture as gold	after a	after a	
		standard	negative TFBS	positive TFBS	
Number of patients in the cohort	142	87	28	80	373
Sensitivity	30 %	30 %	33 %	33 %	62 %
Specificity	90 %	87 %	92 %	95 %	99.7 %
Positive predictive value	25 %	33 %	33 %	20 %	89 %
Negative predictive value	94 %	88 %	92 %	97 %	99 %
Diagnostic odds ratio	5.6	3.7	5,5	8.9	574.4
Positive likelihood ratio	4.2	2.9	4.0	6.3	221.5
Negative likelihood ratio	0.8	0.8	0,7	0.7	0.4

To compare our results with the available literature, we also did a separate calculation in a cohort in which we only included the patients with available microbiological culture samples as a reference ( n=87 ). In this sub-analysis, the NPVs of TPBS and WBC scintigraphy were 93 % and 88 %, respectively.

## 4 Discussion

This study is the first to evaluate the diagnostic value of TPBS and WBC scintigraphy for excluding or diagnosing a low-grade PJI in patients with nonspecific symptoms of their arthroplasty joint (e.g., pain and/or discomfort). The focus on this specific domain of patients resulted in a low prevalence of PJI in our studied cohort (3.5 %). Both TPBS and WBC scintigraphy showed a high negative predictive value, which fits this patient group with a low a priori chance. However, since clinical evaluation already ruled out an infection with a high certainty, a negative nuclear scan showed no clear added value.

TPBS could accurately exclude a low-grade PJI with a NPV of 98 %, which is in accordance with the EBJIS and WAIOT recommendations (McNally et al., 2021; Signore et al., 2019). In 35 % of the TPBSs, an increase in uptake was observed and, as expected, TPBS was not able to differentiate a PJI from other causes, resulting in a PPV of merely 8 %. Even when reducing confounding by analyzing only those scans performed according to the recommendations made in the EBJIS definition (i.e., more than 2 years after arthroplasty for hips and more than 5 years after arthroplasty for knees), 25 %–30 % of TPBSs still showed an increased uptake without an infection being present. In the first 2 years after arthroplasty, 89 of the 149 (60 %) TPBSs were negative versus 131 negative scans out of 191 (69 %) more than 2 years after arthroplasty. So, the common agreement of “the always hot TPBS in the first years after an arthroplasty and therefore not able to rule out a PJI” warrants further evaluation and might even not be true at all. Further prospective research with imaging at fixed time points after arthroplasty is needed to clarify the uptake pattern and the duration of increased uptake for TPBS (Gelderman et al., 2018).

For the WBC scintigraphy, we also observed a high NPV and specificity (96 % and 91 %, respectively), but its sensitivity and PPV were much lower than expected (30 % and 25 %, respectively). In the specific domain of “suspected PJI patients with nonspecific symptoms”, the WBC scintigraphy did not seem to have the diagnostic yield as in other patient categories. This could be due to the low inoculum of bacteria embedded within the biofilm in relation to the relatively low spatial resolution of 8–10 mm of the used SPECT gamma camera system. In these cases, synovial biomarkers and/or tissue biopsies might be a better option in preoperative diagnosis. The disadvantages of the WBC scintigraphy also have to be taken into consideration: that it is time-consuming for both the laboratory technician and the patient, not widely available in all clinics, and not suitable for patients with leukopenia (Glaudemans et al., 2013; Palestro, 2015). Therefore, WBC scintigraphy has a limited role in diagnosing a low-grade PJI in patients with nonspecific symptoms and a low probability of infection but could be used as a “rule-out” test in case of a positive TPBS.

Our results are in accordance with other publications concerning the NPV of both imaging modalities (Verberne et al., 2016, 2017), but most articles demonstrate a higher sensitivity for WBC scintigraphy (Erba et al., 2014). This discrepancy is explained by the chosen domain of our patients. Most of the published studies included patients with a high probability of infection (El Espera et al., 2004; Segura et al., 2004; Pelosi et al., 2004; Love et al., 2004; Pill et al., 2006; Simonsen et al., 2007; Rubello et al., 2008a, b; Love et al., 2009; Glaudemans et al., 2013; Kwee et al., 2013; Kim et al., 2014; Trevail et al., 2016; Auletta et al., 2019; Sengoz et al., 2019; Blanc et al., 2019; Teiler et al., 2020). These studies entail small cohorts of patients (ranging from 19 to 89 patients) and depict a high prevalence of PJI in the studied cohort (ranging from 25 % to 66 %). Our study is a larger cohort ( n=340 ) with a much lower prevalence of PJI (3.5 %). Diaz et al. (2015) performed a critical appraisal of 14 articles investigating the diagnostic accuracy of nuclear imaging for a low-grade PJI, using the QUADAS-2 instruments, and they revealed a high risk of bias in most studies (Diaz-Ledezma et al., 2015; Glaudemans et al., 2016; Diaz-Ledezma et al., 2016; Whiting et al., 2011). Selection bias also seems to be an apparent risk. To illustrate, in a recent study of Blanc et al. (2019), 130 of 298 patients were excluded, because no revision surgery was performed and the prevalence of PJI in this study was as high as 76 %. Additional nuclear diagnostics seems to be redundant in these cases. Furthermore, “doubtful” WBC scintigraphy is not unlikely in patients suspected of a low-grade PJI, and exclusion of these inconclusive scans also creates bias (Lauri et al., 2020). This finding supports strict image acquisition and interpretation criteria.

Our study has major limitations that are mostly related to the retrospective study design (Whiting et al., 2011). The diagnostic protocol was not always strictly followed by the clinicians (e.g., 28 patients received a WBC scintigraphy after an already negative TPBS). It is unclear whether the diagnostic value of nuclear imaging is similar or different in primary arthroplasty and revision surgery, both of which we included. Before 2014 image acquisition and interpretation criteria were collected differently, and as a consequence reported conclusions of the WBC scintigraphies were not in accordance with current existing image acquisition and interpretation criteria, which may have underestimated the results (Van den Wyngaert et al., 2016; Erba et al., 2014; Glaudemans et al., 2013). The low prevalence of PJI is in direct correlation with the sensitivity and PPV and a high negative predictive value. Because of the nature of our chosen domain, not every patient needed revision surgery, and therefore intra-operative cultures as a reference standard were not available in all cases, and 2-year follow-up is chosen as a secondary reference. Nevertheless, this study is based on the reports from that time and therefore reflects clinical practice. This retrospective study is a good starting point for a prospective study including a homogeneous patient population with clearly defined inclusion criteria, strict follow-up and complete data according to newly defined diagnostic criteria (McNally et al., 2021; Parvizi et al., 2018). Furthermore, state-of-the-art image acquisition and interpretation by two independent PJI-dedicated nuclear medicine physicians are important.

Considering the heterogeneity of nuclear imaging protocols and diagnostic pathways applied to our patient populations and the small number of proven PJIs, we are unable to draw any solid scientific conclusions. Our retrospective study suggests that both TPBS and WBC scintigraphy are useful for excluding a low-grade PJI. However, since the chance of finding an infection is very low in this group, regular use of these scans in patients with a low a priori chance of PJI can be omitted. Nevertheless, nuclear imaging could play a role in case of a punctio sicca in which it is not possible to rule out a PJI based on synovial markers. A well-performed prospective study with a homogeneous patient population and clearly defined criteria for diagnosis and interpretation of the scans must be initiated to critically appraise this conclusion.

## Data Availability

The local medical ethical commission does not grant permission for publication of research data and software code.
